# Topological structure of population activity in mouse visual cortex encodes densely sampled stimulus rotations

**DOI:** 10.1016/j.isci.2024.109370

**Published:** 2024-03-04

**Authors:** Kosio Beshkov, Marianne Fyhn, Torkel Hafting, Gaute T. Einevoll

**Affiliations:** 1 Center for Integrative Neuroplasticity, Department of Bioscience, University of Oslo, Oslo, Norway; 2 Institute of Basic Medical Sciences, University of Oslo, Oslo, Norway; 3 Department of Physics, Norwegian University of Life Sciences, As, Norway; 4 Department of Physics, University of Oslo, Oslo, Norway

**Keywords:** Neuroscience, Sensory neuroscience, Cognitive neuroscience

## Abstract

The primary visual cortex is one of the most well understood regions supporting the processing involved in sensory computation. Following the popularization of high-density neural recordings, it has been observed that the activity of large neural populations is often constrained to low dimensional manifolds. In this work, we quantify the structure of such neural manifolds in the visual cortex. We do this by analyzing publicly available two-photon optical recordings of mouse primary visual cortex in response to visual stimuli with a densely sampled rotation angle. Using a geodesic metric along with persistent homology, we discover that population activity in response to such stimuli generates a circular manifold, encoding the angle of rotation. Furthermore, we observe that this circular manifold is expressed differently in subpopulations of neurons with differing orientation and direction selectivity. Finally, we discuss some of the obstacles to reliably retrieving the truthful topology generated by a neural population.

## Highlights

• Mouse primary visual cortex generates circles in response to rotating images
• To extract these circles, we have to use persistent homology with geodesic distances
• Subpopulations of cells generate different circles depending on their selectivity
• Recovering neural topology requires dense sampling of cells and stimuli

## Graphical abstract

**Figure:**
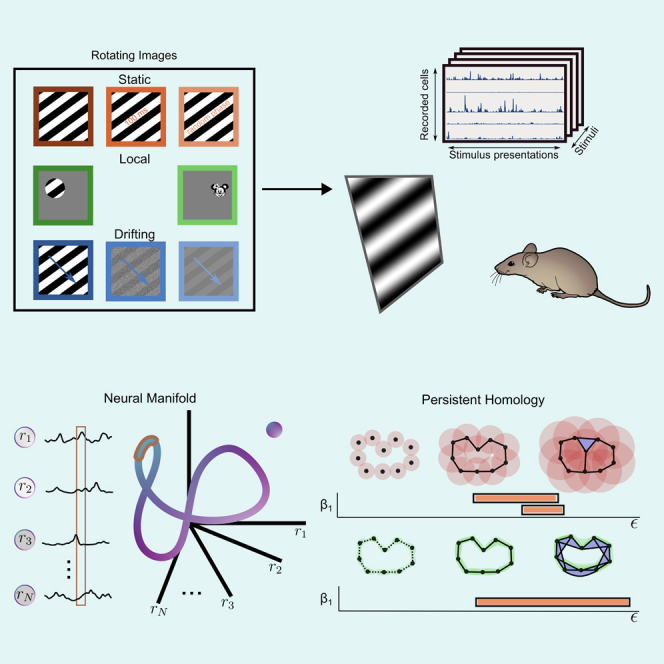

## Introduction

In the past decade, neuroscience has seen a surge in new recording techniques based on both electrophysiological^1^^,^^2^ and optical^3^^,^^4^ methods. While in the past it was only possible to record a few neurons at a time, the techniques of today allow for the simultaneous recording of an immense number of neurons during behavior. Along with the push toward more openness and the increased availability of such large-scale recordings of neural data, this is a truly watershed moment for the development of neuroscience. Such increased accessibility and quality of available data allows the field to transition from generating hypotheses about cognitive function based on single neurons or very small populations to thinking and hypothesizing in terms of neural populations made up of thousands of cells. This methodological change has also resulted in the necessity of new mathematical tools, which are more suited to the analysis of large groups of cells.^5^^,^^6^ Some examples of such tools are *representational similarity analysis*,^7^^,^^8^ *machine learning models*,^9^^,^^10^^,^^11^^,^^12^^,^^13^ and *neural manifolds*.^14^^,^^15^^,^^16^^,^^17^^,^^18^^,^^19^^,^^20^ While such tools seem promising, the results that they provide are more difficult to interpret than single-cell recordings, and their verification can present many conceptual and methodological challenges.

Neural manifolds are a tool that can be used to find structures or features that are not apparent at the level of individual neurons and rely on the idea that computation, dynamics, and the representation of information are mainly realized by large populations of neurons. Neural activity is binned across time or a set of stimulus parameters. The response in each bin can then be interpreted as a point in a high-dimensional vector space in which the neurons form a basis; we will also refer to these points as *states*. This representation of the activity of neural populations allows one to treat neural activity as a manifold, whose structure is connected to the computation implemented by said population. An especially important type of structure to study is the topology^21^ which reflects the way in which different states of activity of the neural population relate to one another.^22^^,^^23^

Studying the topology of neural manifolds has produced many insights. Some examples of this are ring-like manifolds in the mouse head-direction circuit,^24^ toroidal manifolds in rat medial entorhinal cortex,^25^^,^^26^^,^^27^ and the presence of topological organization of place-cell maps.^28^^,^^29^^,^^30^^,^^31^ These examples are all related to navigation or movement, but similar success stories seem to be lacking when it comes to both sensory and higher-order cognitive capacities. While fascinating work on higher-order cognition is ongoing,^32^ understanding it in topological terms is quite a daunting task. Instead, we focus on arguably the most widely studied sensory modality – vision, the analysis of which has become widely popular in light of several publicly available large-scale recordings.^33^^,^^34^^,^^35^

### Previous work on the topology of neural manifolds in vision

There are several papers in which researchers have used dimensionality reduction to study neural manifolds generated in visual tasks.^36^^,^^37^ In the case of sinusoidal gratings, dimensionality reduction has been used to identify a circular manifold.^38^ More advanced dimensionality reduction techniques have been used to decode a toroidal manifold in primary visual cortex induced by the periodic structure of phase and orientation of drifting gratings.^39^^,^^40^ The reduction methods used in these examples rely on visual inspection and remove structural properties of the data, due to the fact that the data have to be projected to a low-dimensional subspace. We address this point further in the STAR methods (Section Dimensionality of the data). On the more theoretical front, there are works proposing that the topological and geometric structure of activity generated by the visual cortex is consistent with that of a sphere^41^ or a Klein bottle.^42^ Finally in a recent publication, Guidolin et al.^43^ investigated the geometry, rather than the topology of visual space by combining spiking metrics^44^ with topological methods and proposed that visual activity is either Euclidean or weakly hyperbolic.

To our knowledge, only two papers have tried to apply topological instead of dimensionality reduction methods in order to quantify the precise topology, rather than geometry, of primary visual cortex in response to visual stimulation. The first paper to do this was^45^ where the authors found a combination of spheres and circles generated by population activity in the primary visual cortex of macaque in a spontaneous and a natural movie condition. This analysis showed proof of principle, but was limited by the fact that only a subset of five cells were used to generate the points ending up in the final analysis. Furthermore, such a result is very difficult to interpret as there is no clear way to extract and conceptually relate the spontaneous and natural movie conditions to their corresponding network states.

The second paper to do this^46^ made use of a publicly available dataset (Allen visual coding neuropixel dataset^34^) to study the topology generated by drifting gratings in various hierarchically organized lower and higher mouse visual regions. In this case, a much larger set of cells were sampled and a distance-based approximation of geodesic distances was used. One would expect that the drifting gratings generate a circular manifold in neural activity. While circles were often observed, especially when the temporal frequency of the drifting gratings was fixed, the analysis also seemed to reveal many other much more complicated topological structures.^46^ However, this work also had a severe limitation, which was that only eight drifting directions were presented.

In our judgment, while both of these works provide an interesting direction for future research, they are still inconclusive as to the precise nature of the topology of neural manifolds generated in visual tasks, even in very simple contexts like the presentation of grating stimuli. Furthermore, both of these analyses make use of electrophysiological recordings, whereas optical imaging methods might be better suited for this type of analysis as they currently include recordings of many more cells.

### Our contributions

In the present paper, we analyze a dataset with up to 50 000 simultaneously recorded neurons from mouse primary visual cortex made openly available in Stringer et al.^35^ Our results show that when using an appropriate metric one can recover the circular topology in neural population activity that is generated by rotating images, which is not the case when using the straight line (Euclidean) metric. This circular topology was observed in the combined activity of all recorded cells, despite the inclusion of cells without particular tuning properties, showing that the angle of rotation of a stimulus is encoded on the global population level and is robust to the inclusion of cells which lack clear tuning properties.

Following this, we show that subpopulations contain different representations of the presented stimuli and that the angle of a rotated stimulus can be recovered more reliably from subpopulations that generate a circular topology. One of these subpopulations is of particular interest as it contains neurons with high orientation selectivity that generate a circular manifold that wraps around on itself and is homeomorphic (has the same topology) to the real projective plane in one dimension. This observation raises interesting questions regarding the topology of neural activity generated by more complicated transformations of three-dimensional objects.

We also show that recovering the expected circular manifolds in mouse V1, requires that both the number of cells and the angles of the presented stimuli are sampled densely. Finally, we discuss three possible obstacles to the identification of topology in the visual system. We propose some potential solutions to these obstacles, which involve precise prescriptions about the type of experiments that are needed to overcome them and push the field further.

## Results

**Figure 1:**
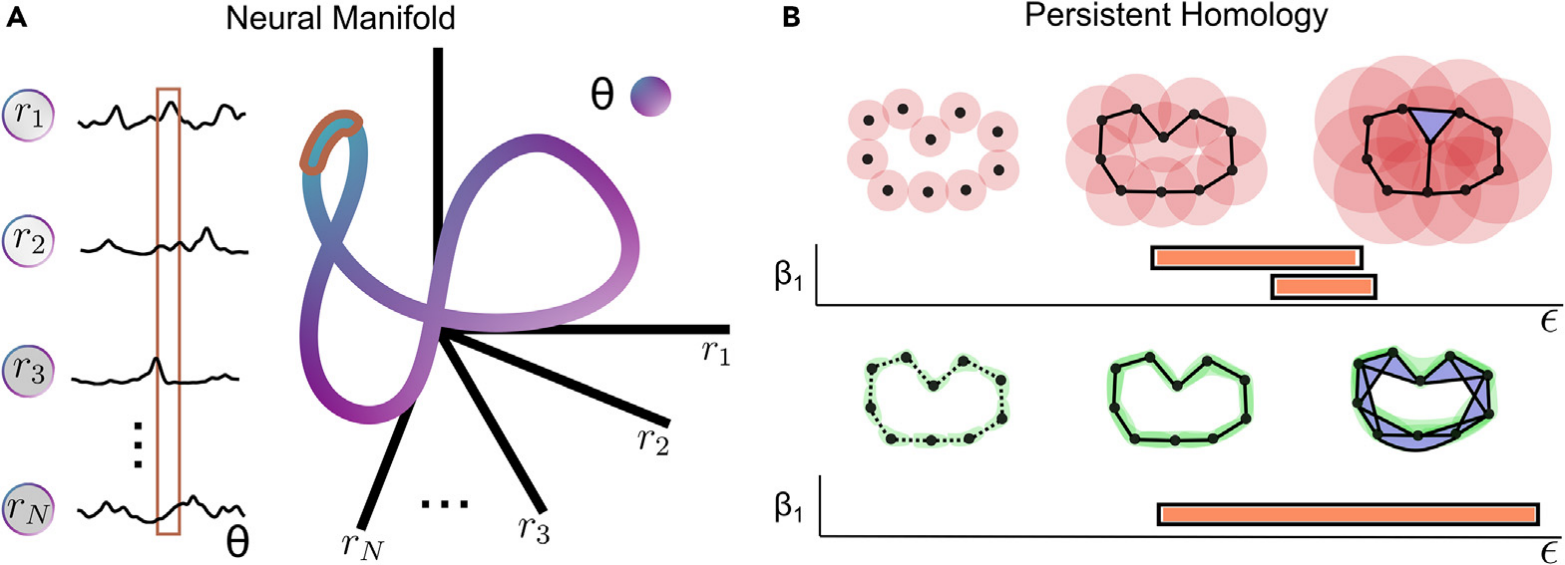
Neural manifolds and persistent (co)homology (A) Illustration of the concept of a *neural manifold*. The responses $r_{1},r_{2},\ldots ,r_{N}$ of a large number of neurons to a stimulus parameter θ (**left**), can be thought of as a point in an *N*-dimensional vector space, spanned by the unit firing rate of each individual neuron. Taking all of these responses together, traces out a *neural manifold* (**right**). Similarly, the responses of a population to a range of stimulus parameters presented at different times during an experiment $\left[\theta_{t},\theta_{t+\Delta t}\right]$, outlined by the brown box over the neural responses, are mapped to a subset of the manifold, outlined by brown. (B) An illustration of the persistent (co)homology algorithm along with a barcode, which is a visual representation of the algorithm’s output in which long bars correspond to highly persistent features. The red circles show how the Rips complex is computed with the Euclidean metric. The green regions show the same for the geodesic metric, with the punctured edges corresponding to the graph generated by the initial choice of k-nearest neighbors (in this case k = 2).

In Figure 1, we attempt to explain the main concepts involved in describing the topology of a neural manifold with the help of persistent (co)homology as well as the extension of the geodesic metric to this method. The fundamental idea is that stimuli can be thought of as coming from a *stimulus manifold* S parametrized by some variables $\theta_{1},\theta_{2},\ldots ,\theta_{M}$. Then, a population of neurons made up of *N* cells, responding to stimuli sampled from the *stimulus manifold*, traces out a surface in an *N*-dimensional vector space spanned by the firing rates of each neuron, denoted by $r_{1},r_{2},\ldots ,r_{N}$. In more precise terms, a neural population applies a mapping Φ, which sends the values of the stimulus parameters to the space of possible population responses $R^{N}$, formally $\Phi :S\to R^{N}$. The surface traced out by this map Φ, is referred to as the *neural manifold*.

There are many ways to determine when two manifolds are different, but here we focus on their topology, or more precisely their (co)homology,^47^ which in simplified terms counts how many holes of different dimensions there are in the manifold. For example, a frisbee is a connected surface and therefore has no holes except one zero-dimensional hole. A more formal way to express the same idea is to say that it has only one non-trivial zeroth (co)homology group (one way to write this with integer coefficients is $H_{0}=Z$ and $H_{i}=0$ ∀i>0), which is known to measure the number of connected components of a space. On the other hand, a hula hoop has two non-trivial (co)homology groups, namely the first and the zeroth ($H_{0}=Z$, $H_{1}=Z$ and $H_{i}=0$ ∀i>0). Therefore, it not only has one connected component, but also has a hole in its center. This concept of holes can be further generalized to any dimension, although the intuition of what a hole is fails to carry over for dimensions higher than two. Often, one prefers to use the Betti numbers $\beta_{i}=\text{rank}\left(H_{i}\right)$ as numerical descriptors of the number of holes instead of thinking directly about the (co)homology groups.

If one has an appropriate representation of a manifold, the (co)homology groups can usually be computed analytically. However, neural data comes in the shape of a point cloud, which is why one has to first define a metric structure to the points. Afterward, one can apply persistent (co)homology 8, to study the topological features of the point cloud. The intuition behind this method is that by growing neighborhoods of size ϵ around each of the points (see Figure 1B), one can obtain an object which describes the topology of the underlying continuous surface in which the topological features, such as the number of connected components and the number of holes, can be computed. Then, the topological features which last for a wide range of ϵ, are called persistent and are thought to correspond to more robust topological features of the data, unrelated to the inherent noisiness in the data generating process.

**Figure 2:**
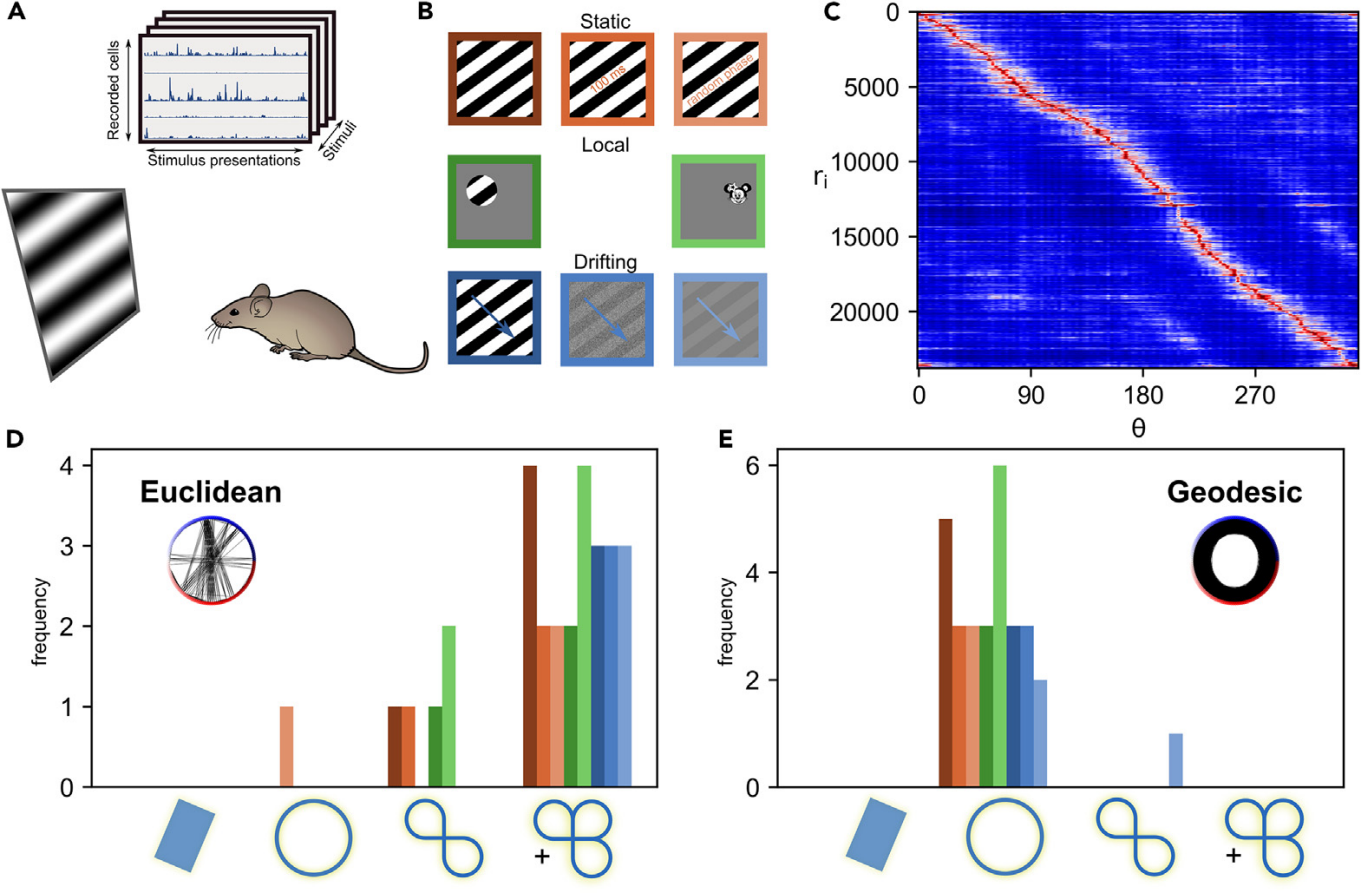
Data processing pipeline and significant (co)homological features (A) Illustration of the obtained data (**top**) and the experimental setup (**bottom**). Stimuli are presented for 750 ms, along with 650 ms of gray screen between them. The response to each stimulus trial is defined as the summed activity during the first 650 ms after stimulus onset. See^35^ for more details about the experiment. The mouse image was sourced from scidraw.io (B) Examples of all stimuli which were presented in the experiment, color coded to match panels (D and E). On the first row: static gratings, short gratings and random phase gratings. On the second row: localized gratings and localized image of Minnie. On the third row: drifting gratings, noisy drifting gratings and low contrast gratings. (C) Neural responses (r) binned by the angle of stimulus rotation and sorted by maximal response angle (θ). n = 23756 neurons from one example mouse. The bin size for an angle was approximately 0.05 radians. (D) Histogram of the identified manifolds (Bonferroni corrected p < 0.0017) across all stimuli and mice, when using the Euclidean metric. The last bin corresponds to cases in which the manifold has more than three holes, or in other words the first Betti number, defined in the STAR methods (Section persistent (co)homology), was larger or equal to three $\beta_{1}\geq 3$. The different colors correspond to the different stimuli shown in panel B. These results show that in most cases neural population activity lives on a manifold with more than three holes. The inset plots show which neural representations of different angles of rotation are considered close by the Euclidean (or the geodesic in panel E) metric. (E) Histogram of the identified manifolds across all stimuli and mice, when using the geodesic metric. According to these results, population activity in response to rotating images lives on a circle.

As mentioned previously, the following results involved the analysis of a publicly available dataset,^35^ which includes multi-plane two-photon recordings of head-fixed mice and contains tens of thousands of neurons at a sampling rate of 3Hz. The mice ($N_{m}=3-6$ depending on the stimulus condition) were presented with a random rotation, uniformly sampled between 0 and 360°, of a stimulus for 750 ms, followed by, on average, a 650 ms gray screen (for more details see^35^). All grating stimuli had a phase of $\frac{\pi }{2}$, which means that gratings which are 180° apart do not overlap and each drifting stimulus drifts in the direction orthogonal to the angle of rotation. A visualization of this experimental pipeline and the main result is shown in Figure 2. For the current analysis, responses were averaged within bins corresponding to ≈0.05 radians of the stimulus angle, shown in Figure 2C. The full preprocessing of this dataset is described in detail in the STAR methods (Section preprocessing and subpopulation selection).

While projections generated by dimensionality reduction algorithms can be informative, they might also obscure important structural information even in the case of low-dimensional manifolds. Such methods have been widely critiqued,^48^^,^^49^ and our analysis does not necessitate such a processing step as both distances and (co)homology can be calculated in the high-dimensional embedding space. Instead of performing dimensionality reduction and showing low-dimensional projections of the high-dimensional manifold, in all figures we show circular graphs, in which an edge is drawn when the distance, computed in the high-dimensional embedding space, between two population states is lower than a chosen threshold.

**Figure 3:**
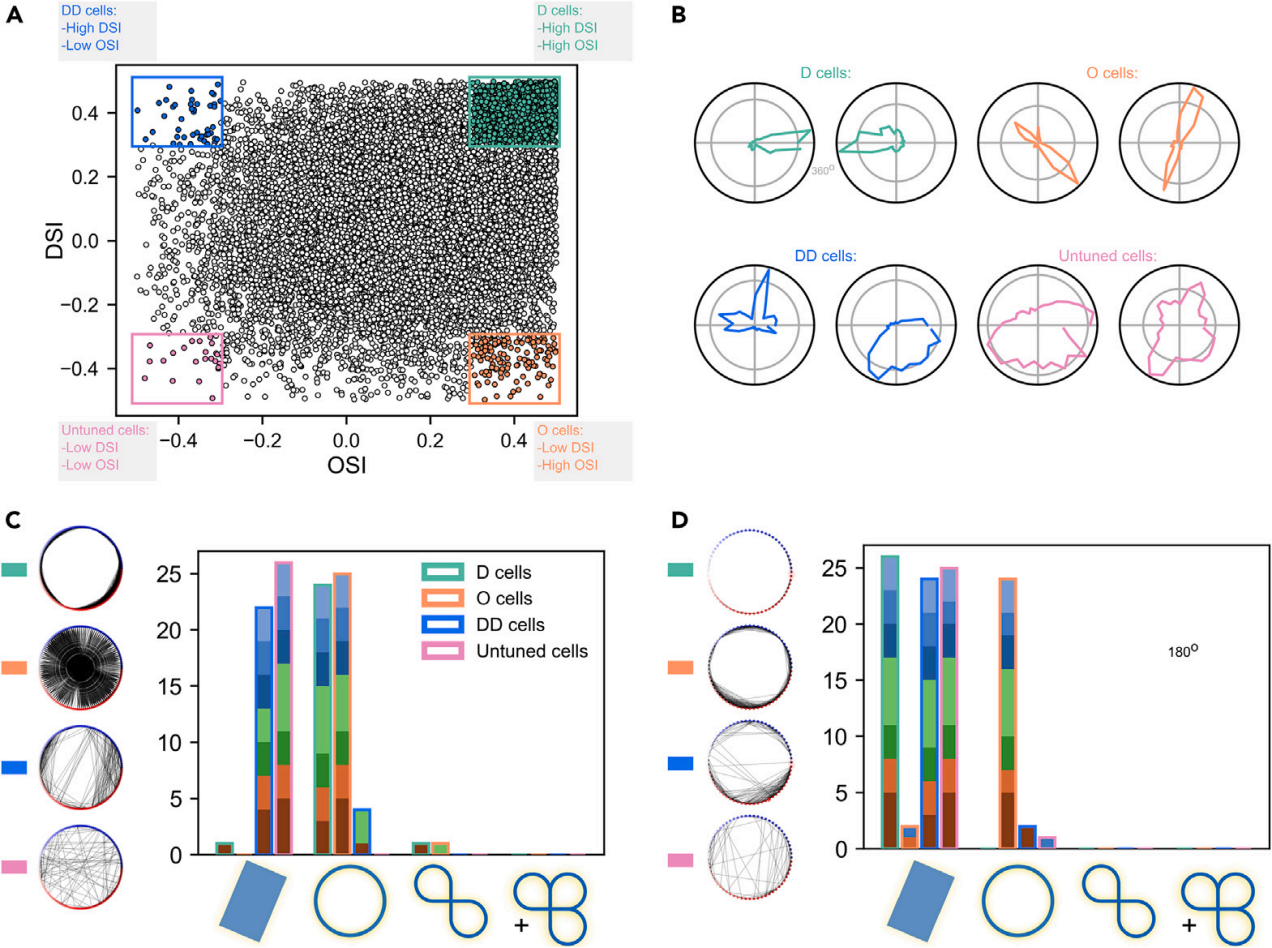
Subpopulations generate different manifolds (A) Scatterplot showing all cells OSI (orientation selectivity index) on the x axis and DSI (directions selectivity index) on the y axis. The four selected subpopulations are highlighted in a different color. D: Direction selective cells (n = 3003), O: Orientation selective (n = 130), DD: Double Direction selective (n = 45), Untuned cells (n = 24). (B) Examples of tuning curves for two cells of each group shown in polar plots. (C) **Left** - example circular graph plots showing the thresholded connectivity between points, corresponding to population responses to stimulus angles, which are organized in a circle and connected by edges if they are closer than the chosen threshold. The threshold was chosen to exemplify the connectivity at which the most robust circle could be observed in the organization of the neural responses. In more precise terms, it is the birth plus one-fourth of the persistence of the largest component of the first (co)homology group. **Right** - histogram of the identified manifolds (Bonferroni corrected p < 0.0017) for each cell group, which correspond to the outline of the bar. The different colors within each bar, denote the different stimuli as shown in Figure 2 panel B. The same analysis, but with the Euclidean metric, can be seen in Figure S3. (D) Same as panel C, except the presented angles were limited to $180^{\circ }$. Furthermore, points at which the manifold fails to connect and complete the circle, are marked by red.

**Figure 4:**
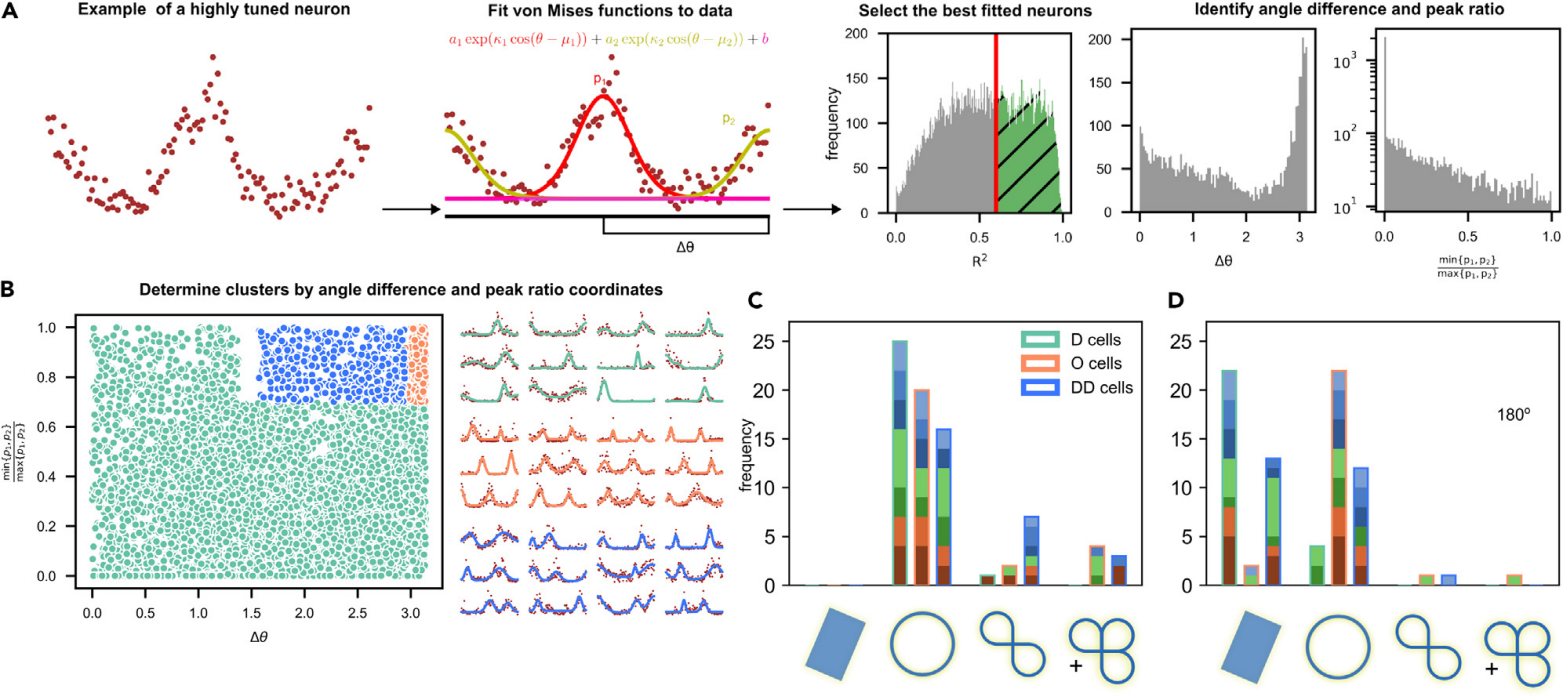
Subpopulations based on a mixture of von Mises functions generate different manifolds (A) Pipeline for cell selection following the fitting of a mixture of von Mises functions. After the fit, cells with $R^{2}>0.6$ are kept. Their peak ratio and angle difference are computed. (B) Cells with an angle difference $\Delta \theta >\frac{15\pi }{16}$ and a peak ratio >0.7 (orange) are classified as O cells. Those with an angle difference $\frac{\pi }{2}<\Delta \theta <\frac{15\pi }{16}$ and a peak ratio >0.7 are classified as DD cells (blue). The remaining cells are classified as D cells (green). (C) Histograms of the identified manifolds for each cell group (Bonferroni corrected p < 0.0017). (D) Same as panel C, except the presented angles were limited to $180^{\circ }$.

**Figure 5:**
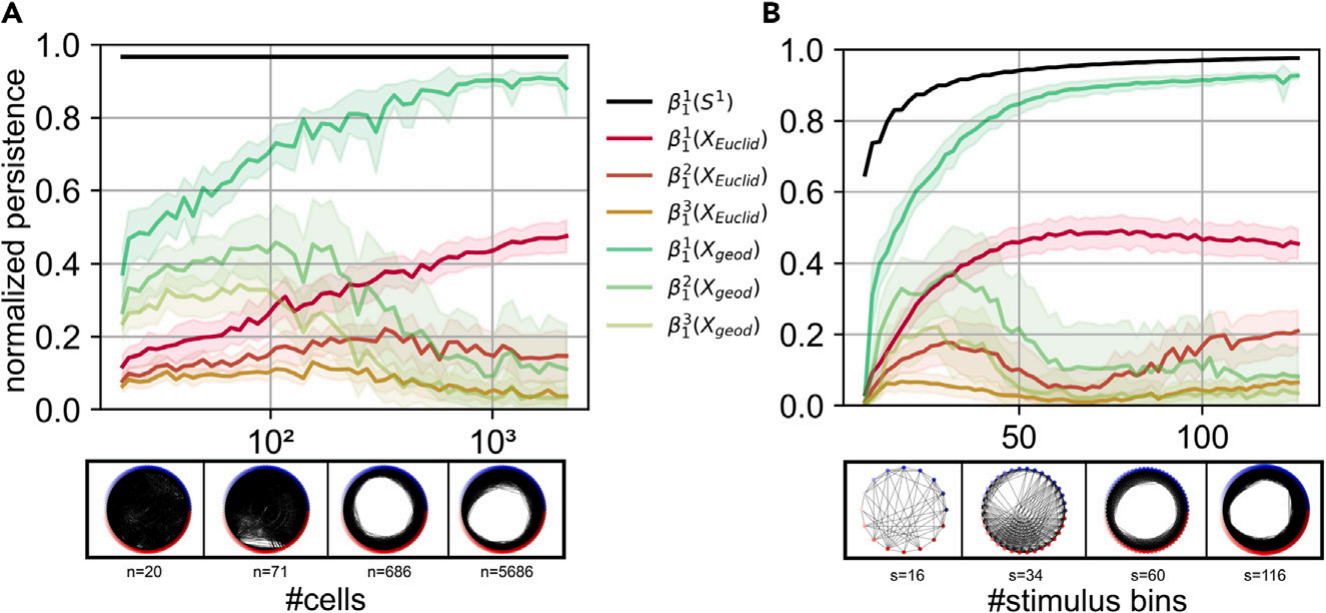
Sufficient sampling is necessary for the recovery of neural topology (A) **Top** - Normalized persistence, calculated by dividing each birth-death pair by the maximum value in the birth-death diagram, of the top three one-dimensional Betti numbers/features, which represent the three most robust circles in the data and are denoted by $\beta_{1}^{1,2,3}$, across all mice and stimuli responding to rotating stimuli whose angles were binned into 90 bins as a function of the number of subsampled cells. The black curve shows the persistence of the first one-dimensional Betti number of 90 points organized in a circle. Green curves correspond to the average feature persistence over mice and stimulus types, along with error intervals showing the standard deviation, after distances were computed using the geodesic metric, whereas the red curves were generated after using the Euclidan metric. **Bottom** - Plots of the thresholded connectivity between state vectors, with *n* being the number of neurons, representing different angles of a stimulus. The threshold was chosen to be half of the maximal distance between points. For all of these plots an approximation of the geodesic metric was used. As one can see, a circular organization appears when a larger amount of neurons is sampled. (B) **Top** - Normalized persistence of the top three features across all mice, with all cells (18639 ± 3869) included, responding to rotating stimuli as a function of the number of bins in which the stimuli were split. **Bottom** - Thresholded connectivity of the activity generated by the full population in response to a stimulus binned into different numbers of angles, with *s* being the number of binned angles.

### Rotating stimuli generate circles in primary visual cortex

In order to identify the topology of the manifolds generated by neural activity, we used an adaptive k-nearest neighbor approximation of geodesics to compute a distance matrix, see STAR methods (Section computing metrics on neural manifolds). Afterward, we computed the persistent (co)homology groups based on these distances. Histograms of the significant first (co)homology groups for all presented stimuli are shown in Figure 2E.

Our results (Figure 2E) point toward the fact that neural population activity in primary visual cortex responding to rotating stimuli lives on a circle in a high-dimensional vector space. This was true not only for symmetric grating-like stimuli, but also for the non-symmetric localized Minnie stimulus. Furthermore, the way that rotations are expressed changes depending on the type of stimulus. For example, a 180° rotation of a static grating corresponds to the same grating but phase shifted by $\frac{\pi }{2}$, whereas a 180° rotation on a drifting grating corresponds to a grating moving in the opposite direction. Thereby, one might think of primary visual cortex as encoding the angle of rotation of a stimulus by mapping it to distinct points on a circle. This result was observed in all stimulus conditions, except for one low-contrast stimulus presentation. In that case our statistical procedure determined that there are in fact two circles (see the barcode plots in Figure S6).

Unlike the results above, which were obtained by using a geodesic metric, use of the Euclidean metric led to much more complex and varied results (Figure 2D). The Euclidean metric failed to identify a consistent and meaningful manifold. This implies that to identify such structure, one requires an approach which is invariant to the high degree of curvature generated by neural activity. In the present case, the geodesic metric managed to deal with this curvature, whereas the Euclidean failed to do so.

### Subpopulations generate different representations of the circle

Contrary to neural circuits involved in higher order cognitive capacities, the responses in the visual system are strongly stimulus-driven.^34^ This fact implies that the manifolds that one observes are less likely to be due to intrinsic attractor dynamics^50^^,^^51^ and more likely to reflect some transformation on the stimulus manifold, which could potentially be so high-dimensional and complex that a low-dimensional attractor model fails to account for said complexity. Given that in this dataset, all stimuli can be parametrized by a single angle parameter, it seems redundant that one needs on the order of $10^{4}$ neurons, to encode a one-dimensional manifold. However, a region such as the primary visual cortex does not have to only encode the angle of rotation, but can be used to employ more complex transformations on the incoming inputs, while a subpopulation encodes the relevant circular variables.

### Subpopulations based on orientation and direction selectivity

This line of thinking leads us to study subpopulations of neurons which exhibit specific tuning properties. To identify these subpopulations, we computed the orientation selectivity index (OSI) and the direction selectivity index (DSI) for each cell (see STAR methods in Section preprocessing and subpopulation selection). Afterward, we identified four groups of cells, based on the most extreme values of these measures ($\left|{\text{OSI}}_{s}\right|>0.3$ and $\left|{\text{DSI}}_{s}\right|>0.3$, with ${\text{OSI}}_{s}=\text{OSI}-E\left[\text{OSI}\right]$ being a standardized selectivity index). One might argue that this way of choosing cells can bias the analysis, due to the fact that a very unbalanced number of cells can fall into each group. To account for this, we also did the same analysis in which we picked the top 100 cells with the most extreme OSI and DSI values (see Figure S4). Additionally, we varied the chosen threshold and observed qualitatively similar results (see Figure S5).

We call the first such group *direction (D)* cells, in reference to the fact that they have a high DSI (>0.3) and a high OSI (>0.3), and are the prototypical example of direction selective cells. We hypothesized that this group would reliably generate a single circular manifold. The second group, *orientation (O)* cells, have a large OSI (>0.3) but a low DSI (<−0.3) and therefore a bimodal tuning curve. This comes about as a result of the fact that if a cell responds in the same way to two rotations which are $180^{\circ }$ apart, then the generated manifold should still be a circle but with opposite points identified. The third identified group had a high DSI (>0.3) and a low OSI (<−0.3). We refer to cells in this group as *double direction (DD)* cells since this combination of orientation and direction selectivity is possible when the cell responds strongly to stimuli with angles of rotation which are 90° apart. This follows from the definition of OSI, which is minimized when the response of a cell to the preferred and orthogonal angles of rotation is the equal. In an ideal case, these neurons should also produce a circle, however if they are very rare or only weakly tuned, they can fail to produce the expected circle. Finally, neurons with both a low OSI (<−0.3) and DSI (<−0.3), show no tuning and are therefore called *untuned*. Since the activity of these neurons is unrelated to the angle of the presented stimulus, it would be very unexpected for them to consistently encode circles.

As can be seen from Figure 3, *D* and *O* cells predominantly generate circles, whereas the other two groups are more likely to generate topologically trivial manifolds (in other words spaces which can be contracted to a point, as they do not have any holes), with *DD* cells rarely generating more complex topological structure. This is likely due to the lack of many such strongly tuned cells in the dataset and when we corrected for unequal sampling by selecting the top 100 such cells, we observed a circular topology in 14 of the datasets as one can see in Figure S4. To verify that *O* cells generate a circle which wraps around on itself, we repeated our analysis while limiting the presented angles to $180^{\circ }$. The histogram in Figure 3D confirms this hypothesis. This can also be seen by the change in connectivity structure of the thresholded circular plots in the same figure.

Additionally, as can be seen in Figure S2, we use ordinary least squares (OLS) regression to show that the angle decoding performance, defined as the coefficient of determination $R^{2}$ on a test set, described further in STAR methods (Section linear decoding analysis), of *O* cells improves significantly when the angles are set {θ→θmodπ}, in other words they obey the relation θ=θ+π, whereas this does not happen in the case of the other subpopulations. It is in stark contrast with *D*, *DD* and *untuned* cells for which performance degrades as a result of this identification, leading to further support for the interpretation that *O* cells truly encode angles on a circle wrapped around on itself. In general, this analysis also supports the idea that cells participating in the generation of the circular manifold are responsible for encoding more information about the angle of rotation of a stimulus.

### Subpopulations based on fitted von Mises functions

We also used a different way to identify subpopulations, exhibiting particular tuning properties, by fitting a mixture of von Mises functions^52^^,^^53^ to each tuning curve and then using the fitted parameters to determine these subpopulations. To do that we look at the peak ratio (defined as the ratio between the smaller and the larger peaks of the two fitted von Mises functions) and the angle difference (defined as the difference between the angles at the two peaks of the fitted cell or $\Delta \theta =\text{arccoscos}\mu_{1}-\mu_{2}$) between well-fitted ($R^{2}>0.6$, the distrbution of $R^{2}$ had mean and standard deviation of 0.45±0.25) tuning curves and end up with three clusters, loosely corresponding to D, O and DD cells in the previous classification, see STAR methods (Section identifying subpopulations from fitted von Mises functions) for more details about the choice of parameters. After selecting cells based on the fitted parameters, we use the raw instead of the fitted tuning curves when calculating persistent (co)homology.

One can clearly see that the D cells identified with this method generate a circle which is lost when the angle of rotation is constrained and the O cells preserve the circle in both situations Figures 4C and 4D. However, likely due to the inclusion of more strongly tuned cells, the DD cells identified in this manner seem to have less noisy tuning and therefore manage to also generate circles some of which remain after constraining the angle of rotation of the stimuli. This subpopulation can potentially be highly heterogeneous and likely includes both D and O-like cells. This explains why it seems to sometimes exhibit behavior similar to both D and O cells.

### Identifying circles in primary visual cortex requires sufficient stimulus sampling

To study the amount of sampling necessary to reliably recover the topology generated by a neural population, we varied the bin size and subsampled different amounts of neurons uniformly without replacement. In Figure 5, we show how much sampling of both cells and stimuli is necessary in order to reliably recover the topology of the circular neural manifold. These results are shown for both the geodesic (green) and Euclidean (red) metrics. As a comparison baseline, we also plotted the persistence of an artificially created perfect circle (black). From these plots one can see that, with the geodesic metric, somewhere on the order of 1000 cells are necessary to reliably recover the topology of the circle, a manifold which is not particularly complicated. When it comes to the sampling of stimulus space, our results indicate that one needs to sample at the very least more than 60 angles to be confident in the estimated topology. Furthermore, in both cases using the Euclidean metric ascribes a large persistence value to not only one, but two one-dimensional features. This happens even when a high number of cells and stimuli is sampled.

It is worth noting that, while the aforementioned numbers are relevant for future experiments of this type, the general amount of sampling necessary to reliably extract the topology of a neural manifold might also depend on other factors. Examples of such factors are the curvature of the neural responses, the dimension of the stimulus space, the complexity of the task, and the cells which a recording method tends to detect. We elaborate on some of these factors in the discussion.

## Discussion

In the present work, we have applied and extended tools from topological data analysis to large-scale neural recordings. We have shown that rotating visual stimuli consistently induce manifolds with a circular topology in mouse primary visual cortex. Furthermore, our analysis shows that subpopulations of cells classified based on traditional tuning-curve statistics, like the OSI and DSI as well as on the fitted parameters of a mixture of von Mises functions, support the generation of circular, projective, and flat manifolds in response to such images. This analysis was only possible due to the availability of large-scale neural recordings^35^ containing not only a dense sampling of both stimuli and cells, but also the choice of an appropriate metric, namely an approximation of the geodesic metric.

This result, and the difficulty of obtaining it, is inconsistent with a pure efficient coding hypothesis,^54^ but aligns well with the research in Stringer et al.,^55^ according to which neural representations in visual cortex maximize a balance between efficiency and smoothness. This balance is expected to produce manifolds which are as highly curved as possible while remaining smooth. This would explain why a dense sampling of stimuli and a metric robust to curvature seem to be so important.

Following this, an important question for future research is what experiments should one perform to capture the full complexity of population activity during the performance of a task? It is crucial to note that, potentially with the exception of highly stable attractor networks, it is unclear how one can begin to disentangle the external input that a task induces in a population from the internal activity generated by said population. Therefore, the topological structure that one finds in a population performing a particular task can not be generalized to other tasks, except when an immense number of possible tasks are presented, which is incredibly difficult, if not practically impossible, to do in an experimental setting. Still one has to wonder whether there might be settings in which task spaces are particularly well suited to be sampled sufficiently densely to obtain an understanding of the system under study.

These are difficult questions and additional work will be needed to answer them fully. In the following discussion section, we try to shine light on some of the obstacles inhibiting our ability to dissect all of this complexity, by understanding the conditions for which one can faithfully recover the correct topology of a neural manifold.

### The sampling obstacle

Previous work by^56^ has proposed the notion of *neural task complexity* (NTC) which bounds the dimensionality of neural activity. A consequence of this work is that besides the obvious dimensionality bound given by the number of recorded neurons, the complexity of the task which an animal is engaged in also plays a fundamental role in determining the properties of the final neural manifold which one can experimentally observe. Taking this point of view, one has to accept that when an experiment is done and neural activity is recorded, the manifold that is extracted is dependent, not only on the number of recorded cells, but also on the structure of the task.

Nevertheless, due to the particular structure of some tasks, the neural manifold generated by a population can be studied. For example, as mentioned before, in tasks related to motor action^15^ and navigation,^24^^,^^25^^,^^26^^,^^28^ very interesting structure has already been extracted. Such tasks have two significant advantages. The first is that they are inherently low-dimensional and as a result sufficiently sampling them is not as difficult. The second advantage is that in such tasks, a very large number of intermediate states are sampled. For example, in the case of head-direction, being in a state characterized by a given angle requires that the animal goes through all intermediate angles. The same is true in the case of movement in a maze, where getting from point A to point B requires the representation of all intermediate states. In other words, due to the nature of these tasks, the stimulus space is always sampled densely.

These two advantages are not present in the case of the visual system, where the dimensionality of visual space is much higher. An estimate of the lower bound on the dimensionality of this space, or rather the space of natural scenes, is given by the dimensionality of ImageNet^57^ which is 43.^58^ Although this is most likely a huge underestimate of the true dimensionality of visual space, it is nevertheless way too high to sample densely in an experimental setting. As was done in this work, this dimensionality problem can often be avoided, by restricting the stimulus space to gratings, which can be parametrized by only a few parameters like orientation, phase and spatial frequency. However, unlike in this work, even such simple experiments often fail to have the second advantage, as often only a few of these stimulus parameters are sampled. As we have shown, even the simplest visual stimulus manifolds, parametrized by a single angle, require the sampling of at least 60 different angles in order to reliably identify topological structure. Therefore, both low-dimensional stimulus manifolds and dense sampling might be necessary.

These problems are not unique to vision, but can also appear in other sensory modalities and higher cognitive functions. Thus, future work trying to understand the neural manifolds generated by neural populations, should carefully consider both the structure and the density with which a task can be sampled and would also benefit from first studying stimulus spaces which can be densely sampled.

### The metric obstacle

After population activity is recorded and a point cloud of population firing-rate vectors is generated, this collection of points is simply a set and there is yet no notion of how these points relate or how distant they are from each other. In order to talk about neural *manifolds*, such a notion is necessary. At first glance, the most parsimonious assumption about these distance relationships is that they should obey the Euclidean metric. As elaborated in Section computing metrics on neural manifolds, this metric does not reflect the possible trajectories between two points, since the transformations which neural populations implement on the initial stimulus manifold are often highly nonlinear^55^^,^^59^^,^^60^ and straight lines between points might correspond to impossible network states.

The most appropriate metric to use would be the geodesic metric,^61^ as it reflects the distance a state has to travel to get to a new state, but calculating it in even the simplest neural network models requires knowing all their parameters and solving a very high-dimensional differential equation. Doing this in actual data is not currently feasible. Nevertheless, such a metric can be approximated using a *k*-nearest neighbor shortest path algorithm, see the STAR methods (Section computing metrics on neural manifolds). A potentially more robust alternative to this approach, based on the UMAP algorithm, has also recently been proposed in.^26^

The geodesic metric is more appropriate to use when one wants to know the topological and geometric properties of the neural manifold extracted in an experiment. However, it is unclear whether the approximated geodesic metric can be used on the neural manifold generated in a different experimental setting. This is because in each experiment the possible population states generated by a task do not exhaust all possible population states in general. Thus, different metrics can be used in tandem to answer different questions regarding *off-* and *on-manifold* perturbations, with the geodesic metric being more suited to detect on-manifold perturbations, whereas the Euclidean metric is appropriate when dealing with off-manifold perturbations. In this case, by an off-manifold perturbation we mean the following: Given an element *s* from a stimulus manifold $S\subset R^{M}$ embedded in M-dimensions, an off-manifold perturbation is a perturbation γ such that s+γ∉S. In contrast, an on-manifold perturbation is a perturbation which does not end up outside the manifold.

The Euclidean metric does not reflect the true distance between stimulus representations. Still, when the stimulus space is restricted, this metric can be used to describe how easy it would be to transform one stimulus into another using off-manifold perturbations. For example, in the data that we have analyzed, the stimulus manifold is given by the set of rotated images S={ρI(x,y)|ρ∈SO(2)}, where SO(2) is the 2D rotation group containing all rotations in two dimensions and I(x,y) is an image. This means that any rotation of an image would be considered an on-manifold perturbation and the geodesic metric should be used to compare the populations responses to such perturbations. However, one could also add noise, scale or even use generative models to interpolate between images in a complicated manner,^62^^,^^63^^,^^64^ which would all impose a different, potentially Euclidean, metric on the data.

Given this task dependence of the metric, future research could focus on generating tasks in which the stimulus manifold is given interesting topological structure and the change in the optimal metric is studied. Additionally, it might be worthwhile to use modeling and simulations of artificial neural networks^65^^,^^66^ in order to understand how network properties relate to the topological properties of the neural manifolds they generate.

### The decoding obstacle

Once a population generates a particular manifold, one might wonder how this encoding of stimulus information is processed by downstream neurons and is typically studied by training a single layer linear decoder.^67^ Since different subpopulations within a region can apply different transformations to a stimulus manifold, there is always the possibility that by analyzing the responses generated by the whole recorded population one misses interesting topological structure in a subpopulation which is processed individually in downstream layers. In the present work, we were able to rely on widely accepted measures of visual tuning curves, with which to identify the subpopulations. However, these measures do not exist for arbitrary tasks and regions and may not be the most appropriate way to understand neural decoding. It is even possible that we have failed to identify more complex and interesting topological structure in the data.

To address such worries, it will be highly beneficial to apply clustering algorithms which are able to extract topologically different and decoding-relevant subpopulations. An approach popular in topological data analysis, known as cohomological decoding,^68^^,^^69^ has already been applied in the context of neuroscience^20^^,^^26^^,^^70^ and future studies can further take advantage of such methods. In the context of grid cells, previous work has made use of clustering methods to identify cells whose activity plays a role in generating a torus^25^ (the neural manifold regularly observed in grid cell recordings). Such cells have even been found to play a more fundamental role in encoding position in artificial recurrent neural network models performing a path integration task, than cells with a high grid score.^71^

### Limitations of the study

Building a theory of neural population function by studying how such populations transform stimulus manifolds into neural manifolds, with potentially different topological or geometric properties, is a young and fascinating research program. Here, we have focused solely on studying topological properties, but this type of analysis is blind to many geometric features of neural manifolds which likely also play an important role in neural computation. Despite the myriad of publications and findings about neural manifolds, pushing this program to more complex and scientifically revelatory settings will not be a trivial task. With the hope of contributing to the development of this research program, we have gone into detail about some of the potential obstacles one might run into when trying to understand neural function in these terms. To address how these obstacles might be overcome, we propose that in order to reliably obtain the true topological structure of a neural manifold, future experiments have to prioritize sampling the stimulus manifold under study densely and choosing an appropriate metric for the analysis of the data. Furthermore, we assert that the field would strongly benefit from topologically inspired clustering algorithms, which will help reveal more fine-grained structure in population activity.

## STAR★Methods

### Key resources table

**Table:** 

REAGENT or RESOURCE	SOURCE	IDENTIFIER
**Deposited data**		

Oriented stimuli dataset	C. Stringer et al.^35^	https://janelia.figshare.com/articles/Recordings_of_20_000_neurons_from_V1_in_response_to_oriented_ stimuli/8279387/3

**Experimental models: Organisms/strains**		

		TetO-GCaMP6s x Camk2a-tTA mice JAX RRID:IMSR_JAX:024742 and RRID:IMSR_JAX:007004

**Software and algorithms**		

Ripser 0.6.1	N. Saul and C. Traile.^84^	https://ripser.scikit-tda.org/en/latest/Matplotlib 3.2.2
https://matplotlib.org/Scipy 1.7.3	P. Virtanen^72^	https://scipy.org/Scikit-learn1.2.0
https://scikit-learn.org/stable/Networkx 2.8.8	A. Hagberg et al.^77^	https://networkx.org/Original code: Geodesic approximation (current study) https://github.com/KBeshkov/NeuralHomology

### Resource availability

#### Lead contact

Questions regarding our work can be directed to the lead contact, Kosio Beshkov at kosio.neuro@gmail.com.

#### Materials availability

This study did not generate new unique reagents.

#### Data and code availability

• For the present study we used openly available data,^35^ which can be found at https://doi.org/10.25378/janelia.8279387.v3.
• All original code has been deposited at https://github.com/KBeshkov/NeuralHomology and is publicly available as of the date of publication. DOIs are listed in the key resources table.
• Any additional information required to reanalyze the data reported in this paper is available from the lead contact upon request.

### Experimental model and study participant details

Our analysis relied on openly available recordings of female and male mice ranging between 2 and 12 months of age. No new samples were collected for the present study.

### Method details

We obtained the data from Pachitariu et al.^33^ The recording and preprocessing of this dataset is described in detail by the authors in.^35^ Here we describe the stimuli along with all additional processing we applied to study topological structure.

#### Stimulus description

All presented stimuli can be characterized by a single variable which is their orientation (angle of rotation). Every other variable like contrast, location, spatial frequency and presentation time was fixed within each stimulus type.

#### Static

Static gratings, like most other stimuli, were presented for 750 ms, with a spatial frequency of 0.05 cycles per degree and had a fixed phase. Given the fact that the phase of these gratings is fixed at $\frac{\pi }{2}$, orientations which are 180° apart are nonoverlapping, whereas orientations which are 360° apart generate the same grating. Therefore one can expect that these stimuli will generate a circle in neural activity.

#### Short

Short gratings had the same properties as static ones, with the exception that they were only presented for 100 ms. Without an additional hypothesis about how long it takes for neurons to respond to a stimulus, one would expect that these stimuli would generate the same manifold.

#### Random phase

For these stimuli the phase was randomly sampled, which leads to gratings which are 180° apart being much more similar. This effectively means that orientation is restricted between 0 and 180°, which implies that as a function of orientation up to 360°, these stimuli should generate a circle which wraps around on itself. One more important thing to note is that both unimodal as well as bimodal (or what we call O cells) neurons will respond in the same way to these stimuli and we have therefore excluded them from the subpopulation analysis.

#### Local

Local gratings were presented for the same duration and covered 30 degrees of visual space, in which only one white and one black subfield were visible. The rest of the screen was gray. In this case one would expect that these stimuli would also generate a circle. However, this circle would be mostly generated by cells whose receptive fields overlap with the stimulus, whereas other cells will only add noisy baseline activity in non-responsive dimensions.

#### Minnie

The Minnie stimulus is by far the most unique one as it is more complex and lacks the obvious rotational symmetries of the other stimuli. It was similarly localized to 30 degrees of the visual field and was rotated around its center. It is difficult to predict the exact manifold that such a stimulus will generate. For example, due to rotation invariance, rotating a white circle should generate a single point, whereas rotating a star with five rays should generate a circle which wraps 5 times on itself. Since the rotational symmetries of arbitrary stimuli are unknown it is difficult to be sure what manifold will be generated by a neural population. One thing that is certainly known is that if only 360° rotations leave the object invariant then it is likely that the generated manifold will be a circle. Exploring how symmetries of such objects are represented by neural populations is a difficult problem and an interesting direction for future research, as it can be used to confirm whether the visual system preserves or changes the topological structure of a stimulus set.

#### Drifting

Drifting gratings were presented with a temporal frequency of 2 Hz. In this case, for each orientation of a grating there is a single corresponding drifting direction, which is orthogonal to the orientation. With this in mind one would expect that these stimuli also generate a circle since, due to the correlation between orientation and drifting direction, there is a single periodic variable characterizing the manifold.

#### Noisy

Noisy gratings were also drifting and had a contrast of 5%. Depending on the amount of noise, one would predict that these stimuli either generate a circle (if the noise is not too high and the contrast is not too low), or a solid non-isotropic (due to the fact that baseline activity is not constant across neurons) ball of dimension equal to the number of neurons.

#### Low contrast

Just like the noisy gratings, low contrast gratings had a contrast of 5%. Similarly one would expect them to generate a circle, except if the contrast is very low.

It is important to keep in mind that while there is one single angle parameter which characterizes each stimulus condition, the meaning of this parameter is not the same across different conditions. For example a 180° rotation corresponds to a phase offset in static gratings, but it corresponds to a direction of motion in drifting gratings.

#### Preprocessing and subpopulation selection

In order to obtain a topologically robust point cloud, we split the angles of each image into 126 bins, each one of size ≈0.05 radians, covering the full range of [0,360) degrees and averaged all population responses in these bins. This left us with a 126 × #neurons matrix for each image/animal condition, to which we could then apply persistent homology.

In order to extract the four subpopulations which ended up in our final analysis, we first computed the OSI and DSI as,

(Equation 1) $$\text{OSI}=\frac{r_{pref}-r_{orth}}{r_{pref}+r_{orth}},\text{DSI}=\frac{r_{pref}-r_{opp}}{r_{pref}+r_{opp}}\text{.}$$

With $r_{pref},r_{orth},r_{opp}$ being the responses at preferred (the angle at which a cell responds maximally), orthogonal (the angle orthogonal to the preferred angle) and opposite (the angle 180° from the preferred angle) directions respectively. We further standardized these measures (${\text{OSI}}_{s}$ and ${\text{DSI}}_{s}$) to be in the range ${\left[-0.5,0.5\right]}^{2}=\left[-0.5,0.5\right]\times \left[-0.5,0.5\right]$ by subtracting their mean. For the analysis shown in Figure 3, we then only considered strongly selective cells in the union of subsets corresponding to different cell classes ${\left[-0.5,-0.3\right]}^{2}\cup \left(\left[-0.5,-0.3\right]\times \left[0.3,0.5\right]\right)\cup \left(\left[0.3,0.5\right]\times \left[-0.5,-0.3\right]\right)\cup {\left[0.3,0.5\right]}^{2}$. To make sure that this analysis was not strongly dependent on choosing 0.3 as a threshold we repeated the analysis for choices of 0.35 and 0.25 and obtained qualitatively identical results, see Figure S5.

For the decoding analysis, described in the next section, one might argue that to fairly compare decoding performance of the different cell classes, each class should contain the same number of neurons. To verify whether this could make a difference, we sorted the neurons into four groups, depending on which quadrant they occupied in OSI_s_ × ${\text{DSI}}_{s}$ coordinates. After this we sorted them by their l1 norm in this coordinate system.

(Equation 2) $${\left\|x\right\|}_{1}=\left|\text{OSI}\left(x\right)\right|+\left|\text{DSI}\left(x\right)\right|\text{.}$$

After this we selected the top 100 cells for further analysis (shown in Figure S4).

#### Identifying subpopulations from fitted von mises functions

In order to obtain a more descriptive classification of neural subpopulations, we fit the following mixture of von Mises functions to each neurons tunning curve:

(Equation 3) $$f\left(\theta \right)=f_{1}\left(\theta \right)+f_{2}\left(\theta \right)+b=a_{1}\;\exp\left(\kappa_{1}\;\cos\left(\theta -\mu_{1}\right)\right)+a_{2}\;\exp\left(\kappa_{2}\;\cos\left(\theta -\mu_{2}\right)\right)+b\text{.}$$

To perform the fit we used the curve_fit function from the scipy package,^72^ with the Trust Region Reflective (TRF) method^73^ with bounds $a_{1},a_{2},\kappa_{1},\kappa_{2},b\in [0,\inf)$ and $\mu_{1},\mu_{2}\in \left[-\pi ,\pi \right]$. After this we only worked with fits for which $R^{2}>0.6$. To classify subpopulations we use the fitted parameters of each cell to compute two statistics, the angle difference $\Delta \theta =\arccos\left(\cos\left(\mu_{1}-\mu_{2}\right)\right)$ and the peak ratio $P=\frac{\min\left\{p_{1},p_{2}\right\}}{\max\left\{p_{1},p_{2}\right\}}$, where $p_{i}=f_{i}\left(\mu_{i}\right)+b$. The angle difference is informative for whether a cell is bimodal, but also for the different angles which it encodes. The peak ratio is needed to exclude cases in which the angle difference indicates the presence of bimodal tuning but one of the peaks is very small.

Using these statistics, we identified three types of subpopulations corresponding to bimodal tuning curves encoding stimuli with a different angle difference. The first such subpopulation consisted of cells with $\Delta \theta <\frac{\pi }{2}$ or P<0.7 and corresponds to cells which are for the most part unimodal and conceptually match the D cells identified with the selectivity classification. The second subpopulation corresponds to bimodal neurons with P>0.7 and $\Delta \theta >\frac{15\pi }{16}$. Essentially the goal for this subpopulation was to find bimodal cells whose peaks are opposite from each other, thereby matching O cells. The final subpopulation had P>0.7 and $\Delta \theta \in \left(\frac{\pi }{2},\frac{15\pi }{16}\right)$, which included many examples of bimodal tuning curves with different spacing between their peaks. Such curves are in a sense doubly tuned, so they conceptually match the DD cells identified from the previous analysis. It is important to make note of the fact that these parameter choices were chosen in order to separate subpopulations into classes conceptually related to the classification based on the OSI and the DSI. Still, they are quite arbitrary and different choices could potentially lead to the discovery of groupings generating other topological features.

#### Dimensionality of the data

One might argue that when the set of visual stimuli is simple enough, dimensionality reduction methods are sufficient to study the structure of neural activity directly by visualizing it, since only a few components are necessary to explain a large portion of the variance. However, visual responses to simple stimuli, while intrinsically low dimensional, seem to posses a high degree of curvature, and as a result it is not clear that such preprocessing steps are always justified.

In the presently analyzed data we z-scored each binned dataset and applied principal component analysis (PCA) to it. We observed that the first 2 components managed to explain 36% ± 8% of the variance on average, whereas using the top 10 components explained 79% ± 5%. This supported the idea that one should prefer a dimensionality-free topological approach.

One might still object, that while linear methods might not be appropriate, nonlinear techniques like Isomap,^74^ t-SNE^75^ and UMAP^76^ should still provide a reliable low-dimensional representation of the data. For one, these methods are much harder to understand and verify, and that is enough of a reason to avoid them when less aggressive alternatives are on the table. However even ignoring that, they sometimes failed to visually reproduce the correct manifold structure by generating more than one circle on several datasets. Some visual examples are given in Figure S1. It is worth noting that these failures are quite rare especially when using Isomap.

Instead, for the purpose of visualization of the neural manifolds, we opted to use thresholded circular graphs, in which one creates a graph by considering each point in a point cloud as a vertex and drawing an edge between two points whenever the true distance between them in the high-dimensional space is lower than a specified threshold. Afterward the graph is embedded in two dimensions and organized in a circle, which is especially convenient when one is exploring circular manifolds. Given this organization, if a circle is present then the edges only connect neighboring vertices and do not cross over to the other side. The presence of edges crossing over hints that the manifold is not circular. The big advantage of this approach is that one can directly see the connectivity in circles in which opposite points are identified, which happens to be the case in *O* cells and can be directly seen in Figure 3C. The threshold was chosen to be one-fourth of the persistence of the biggest circular features plus its birth, which is when the circle appears clearly while avoiding unnecessary clutter. This visualization procedure was done using the Networkx Python package.^77^

#### Linear decoding analysis

In order to compare the decoding performance across different subpopulations we randomly split all response/angle pairs into a training (80%) and a test (20%) set. Then we performed linear regression in order to predict the angle of each stimulus from the subpopulation response.

To evaluate the performance of the linear decoder, we evaluated the coefficient of determination $R^{2}$, given by the formula,

(Equation 4) $$R^{2}=1-\frac{\sum {\left(y_{i}-f\left(y_{i}\right)\right)}^{2}}{\sum {\left(y_{i}-\bar{y}\right)}^{2}}\text{,}$$

where $x_{i}$ and $y_{i}$ are the input and target points to a statistical model applying the map f(⋅). To determine significance we used a Bonferroni-corrected Wilcoxon rank-sum^78^ test on each pair of subpopulation $R^{2}$ distributions. While non-linear decoders can surely improve the decoding performance, they introduce additional assumptions,^67^ which only overcomplicate our analysis and are therefore outside of the scope of this work.

#### Computing metrics on neural manifolds

Metrics, also known as distance functions, are a way in which to compute the distance between two points in any space. The most famous such metric is the flat/Euclidean metric, which is simply given by the expression,

(Equation 5) $$d\left(x,y\right)=\sqrt{\sum_{i}{\left(x_{i}-y_{i}\right)}^{2}}\text{.}$$

This is a good metric when neural activity lives in flat space, but in the case of a more complicated manifold with curvature, a geodesic metric is more appropriate. This type of distance is given by the expression,

(Equation 6) $$d\left(x,y\right)=\min_{\gamma }\int_{0}^{1}g\left(\dot{\gamma }\left(t\right),\dot{\gamma }\left(t\right)\right)dt\text{.}$$

Where γ(t) is any curve connecting *x* and *y* on the manifold, and *g* is the metric tensor, for more information see.^61^ While this is the “correct” metric, it is only possible to obtain an expression for it if one knows the precise expression which generates neural activity and even then solving this optimization problem is non-trivial. In the case of neural recordings, such an expression definitely does not exist, therefore we opt for a numeric approach with which to approximate these geodesics. This approach has previously been applied in,^46^ with the slight difference that the nearest neighbor graph was constructed by fixing a distance threshold instead of finding the *k*-nearest neighbors, and is similar to newer developments in which the UMAP algorithm has been adapted to approximate geodesics.^26^

The geodesics are approximated by the following stepwise algorithm.

(1) Given a point cloud *X* embedded in an n-dimensional space $R^{n}$, choose a number of neighbors *k* and construct the k-nearest neighbors graph.
(2) Compute the distance between each pair of points by calculating the shortest path on the graph between them.
(3) (*adaptive - optional*) If k was chosen too low, there might be disconnected clusters of points between which the distances are infinite. To fix this, one can either split the clusters and analyze them separately or increase *k* until there is a path between any two points. To achieve the second option one can store the computed distances and increase k→k+1. Then any distances that were previously impossible to calculate can be recomputed until no such distances remain.

For all data analysis we chose a small k=4 and used the adaptive version of the algorithm to deal with outliers.

#### Persistent (co)homology

*Topological data analysis*, is a rapidly growing field which adapts the computational methods developed for the classification of topological spaces in the late 19th century to the data-rich world of today.^79^^,^^80^^,^^81^ One of the most popular tools from this field is *persistent (co)homology*, which characterizes the structure in a point cloud by counting the holes that appear and disappear in it on different spatial scales.^82^^,^^83^

These computations involve several steps.

1 Given a point cloud *X* embedded in an n-dimensional metric space $\left(R^{n},d\right)$, construct the *Vietoris-Rips complex* $V_{\epsilon }\left(X\right)$ by adding all subsets of points which are at a distance smaller or equal to ϵ from each other: $V_{\epsilon }\left(X\right)=\left\{\sigma :d\left(x_{i},x_{j}\right)\leq \epsilon ,\forall x_{i},x_{j}\in \sigma \right\}$. Each σ with k elements in this set corresponds to a k-simplex.
2 Associate the simplices $S_{n}$ of each dimension to a vector space $C_{n}$ over a field of coefficients (in this case the field Z/2Z is used).
3 Equip each such vector space with *boundary maps* $\partial_{n}$, with the property that $\partial_{n}\circ \partial_{n+1}=0$ on any element of any $C_{n}$. The collection of these vector spaces along with the corresponding boundary maps is called a chain complex. This chain complex $C_{*}$ is visualized with arrows, corresponding to boundary maps, in the following way,

(Equation 7) $$0\leftarrow C_{0}\leftarrow C_{1}\leftarrow C_{2}\leftarrow \ldots$$

(1) If ϵ<δ, there exists an inclusion $\iota :V_{\epsilon }\left(X\right)\to V_{\delta }\left(X\right)$. This inclusion induces an ordering on the generated simplicial complexes and therefore also an ordering on the chain complexes $C_{*}^{0}\to C_{*}^{1}\to C_{*}^{2}\to \ldots$, where the upper indices correspond to increasing non-negative values of ϵ and the arrows correspond to the induced maps $\iota_{*}^{i}:C_{*}^{i}\to C_{*}^{i+1}$. This sequence of chain complexes is called a *persistence complex.* It can be visualized like this,

**Figure:**
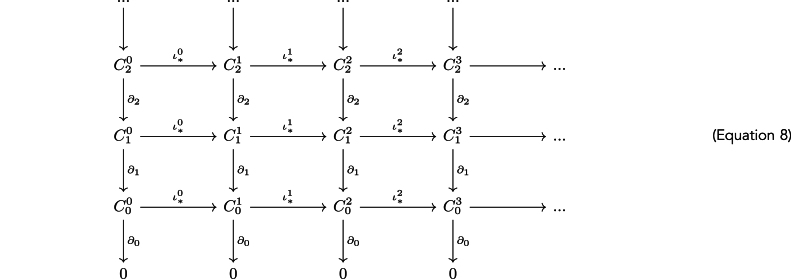
(2) Given this construction the next step is to compute the (co)Homology groups associated to the complex at each scale ϵ. This is done by computing $H_{i}\left(V_{\epsilon },Z/2Z\right)=\mathrm{Ker}\left(\partial_{n}\right)/Im\left(\partial_{n+1}\right)$. The ranks of the (co)homology groups are known as the Betti numbers, defined by $\beta_{i}=\text{rank}\left(H_{i}\left(V_{\epsilon },Z/2Z\right)\right)$, are also computed as a more intuitive summary of the (co)homology groups.
(3) Finally, the ϵ values at which new (co)homology classes appear are called the births and the values at which they disappear are called deaths. A barcode is the collection of birth-death pairs ${\left\{\left(b_{i},d_{i}\right)\right\}}_{i}$ and large intervals in this collection are interpreted as significant topological features unrelated to noise. From each such interval, the persistence of its corresponding feature is defined as $p_{i}=d_{i}-p_{i}$

To perform these computations we made use of the openly available Python library *Ripser*.^84^^,^^85^ For a more rigorous treatment of algebraic topology see.^47^^,^^86^

### Quantification and statistical analysis

#### Statistical testing for persistent features

While the question of when a topological feature can be considered significant is still an active research area,^87^^,^^88^ here we stuck to an approach which is highly similar to the ones used in previous applications of topological data analysis to neuroscience. Essentially the idea is that given a point cloud, one can shuffle the responses of each neuron independently and take the feature with maximal persistence $\max\left\{p_{i}\right\}$ from a normalized persistence diagram. By repeating this enough times a distribution of the maximal components is generated, then one can compare the persistent topological features found in real data with the ones occurring in randomly shuffled data.

We only report the results of these statistical tests on one-dimensional topological features, since higher-dimensional features are both unexpected when the input manifold is one-dimensional and we failed to find any such persistent components. For all statistical tests we used 1000 shuffles and a Bonferroni-corrected p value of 0.0017. Therefore, a topological feature was determined to be significant when it was larger than the 99.83th percentile of the shuffle distribution.
