# Supplementary material for: Topological structure of population activity in mouse visual cortex encodes densely sampled stimulus rotations
Source: iScience. 2024 Mar 4;27(4):109370. doi: 10.1016/j.isci.2024.109370 (PMC10959658; doi:10.1016/j.isci.2024.109370)
Supplement: Document S1. Figures S1–S6 [file mmc1.pdf]

iScience, Volume *27*

Supplemental information

Topological structure of population activity in mouse visual cortex encodes densely sampled stimulus rotations

Kosio Beshkov, Marianne Fyhn, Torkel Hafting, and Gaute T. Einevoll

Supplementary Information

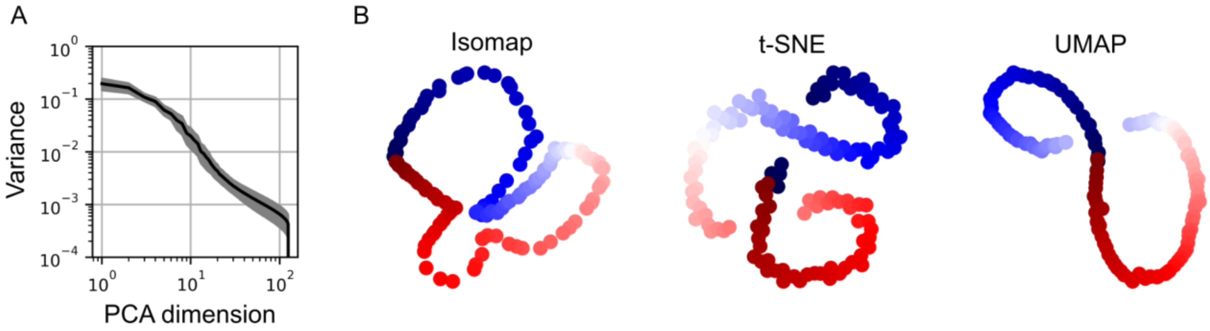

Figure 1: **Performance of dimensionality reduction techniques as discussed in the STAR Methods.**
A) Explained variance by principal components averaged over datasets.
B) Illustration of manifolds generated by different dimensionality reduction techniques, which are distorted from the topologically estimated geometry.

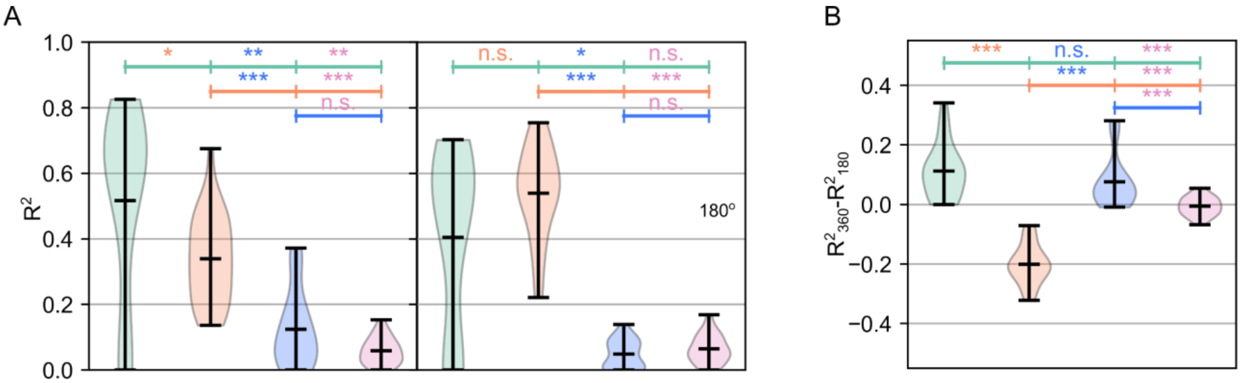

Figure 2: **Decoding performance with ordinary least squares regression using the neural subpopulations shown in Figure 3.**
A) Ordinary least-squares decoding performance of the different subsets of cells. To determine significance we used a Bonferroni-corrected Wilcoxon rank-sum test. *D* and *O* cells showed good performance, whereas less strongly tuned cells under-performed in decoding the angle at which a stimulus was presented. The left panel shows predictions for the full 360° of directions, whereas in the right panel the directions were recomputed as $\theta = \theta$ mod 180°.
B) The difference between the predictions showed that only orientation selective cells exhibit a significant improvement in their performance when it came to recomputed stimuli.

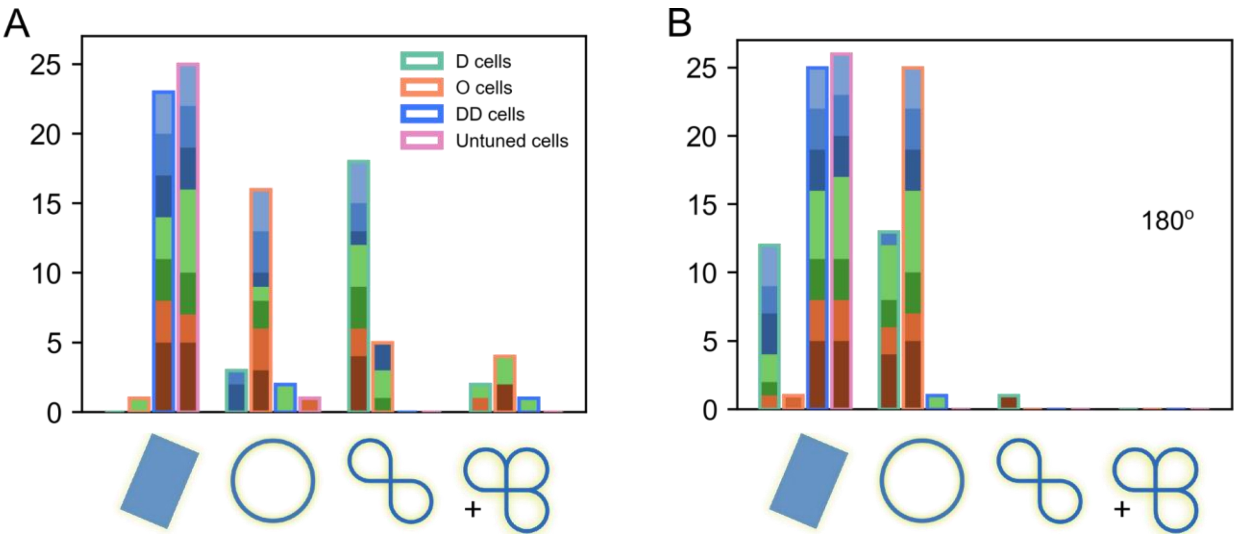

Figure 3: **(Co)homological features extracted with the Euclidean metric using the neural subpopulations shown in Figure 3.**

A) Histogram of the identified manifolds across all stimuli using the Euclidean metric. While circular features are identified, the high curvature of the neural responses leads to the appearance of too many holes.

B) Same as A, except the presented angles were limited to 180°. The differences between A and B make the same point as in Figure 2, namely O cells encode the rotations on a circle even when the presented angles are restricted. However, the identified topology is much less reliable.

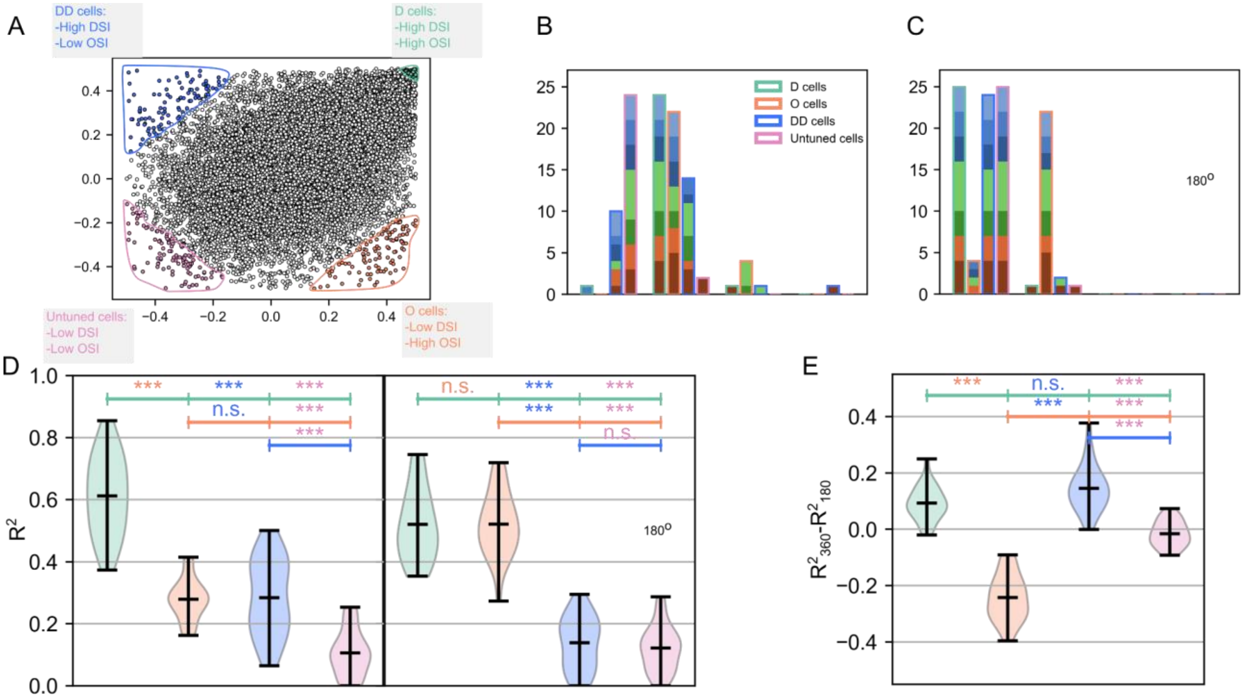

Figure 4: **Subpopulations of the top 100 strongest tuning cells show similar features to the subpopulations shown in Figure 3.**

A) Scatter plot showing the identified cell groups.

B) Histogram of the identified manifolds for each cell group.

C) Same as panel B, except the presented angles were limited to 180°.

D) Ordinary least squares decoding performance of the different subsets of cells. To determine significance we used a Bonferroni-corrected Wilcoxon rank-sum test. DO and O cells showed good performance, whereas less strongly tuned cells under-performed. The left panel shows predictions for the full 360° of directions, whereas in the right panel the directions were recomputed as $\theta = \theta$ mod 180°.

E) The difference between the predictions showed that only O cells exhibit a significant improvement in their performance when it came to recomputed stimuli.

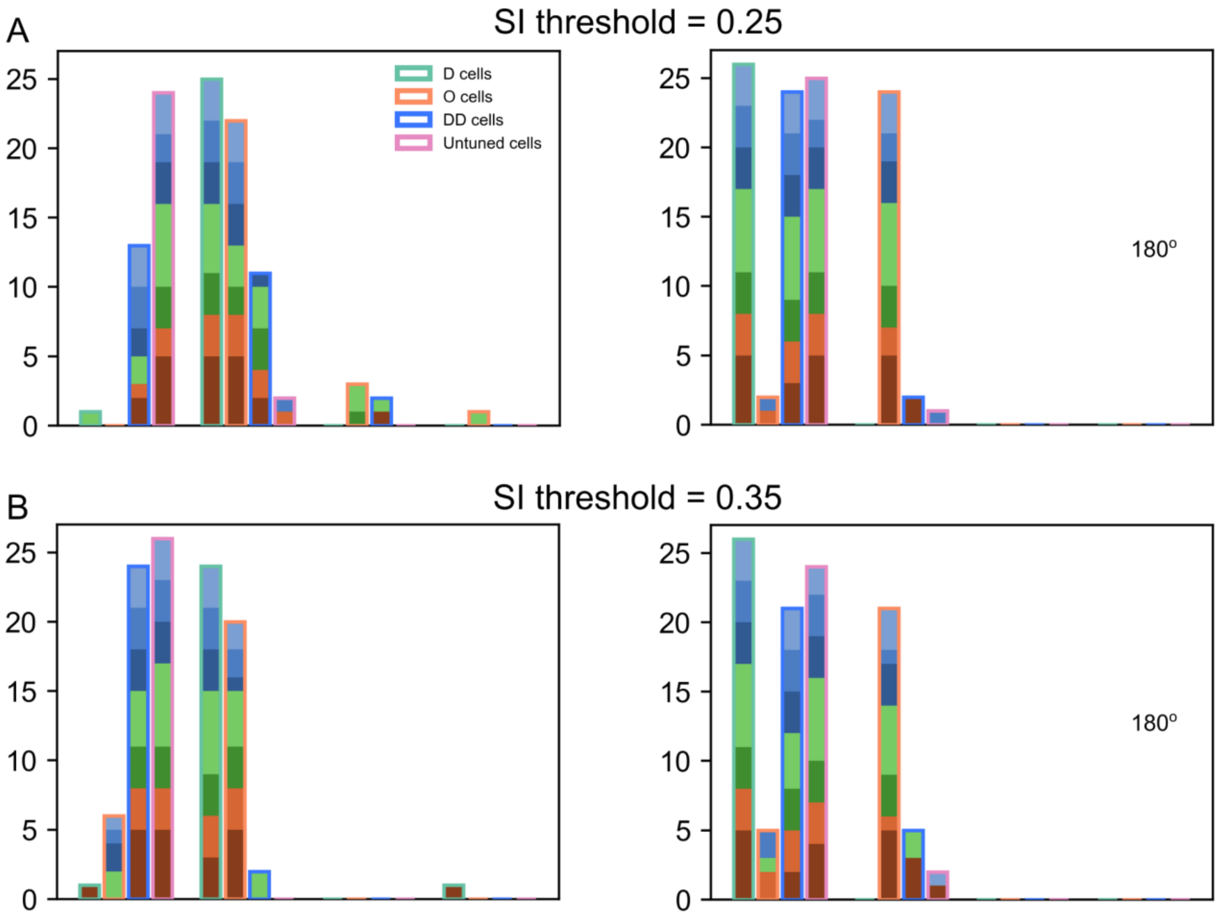

Figure 5: **Different SI thresholds lead to qualitatively similar results as thos with the subpopulations shown in Figure 3.**

A) The identified topological features for an SI threshold of 0.25.

B) The identified topological features for an SI threshold of 0.35.

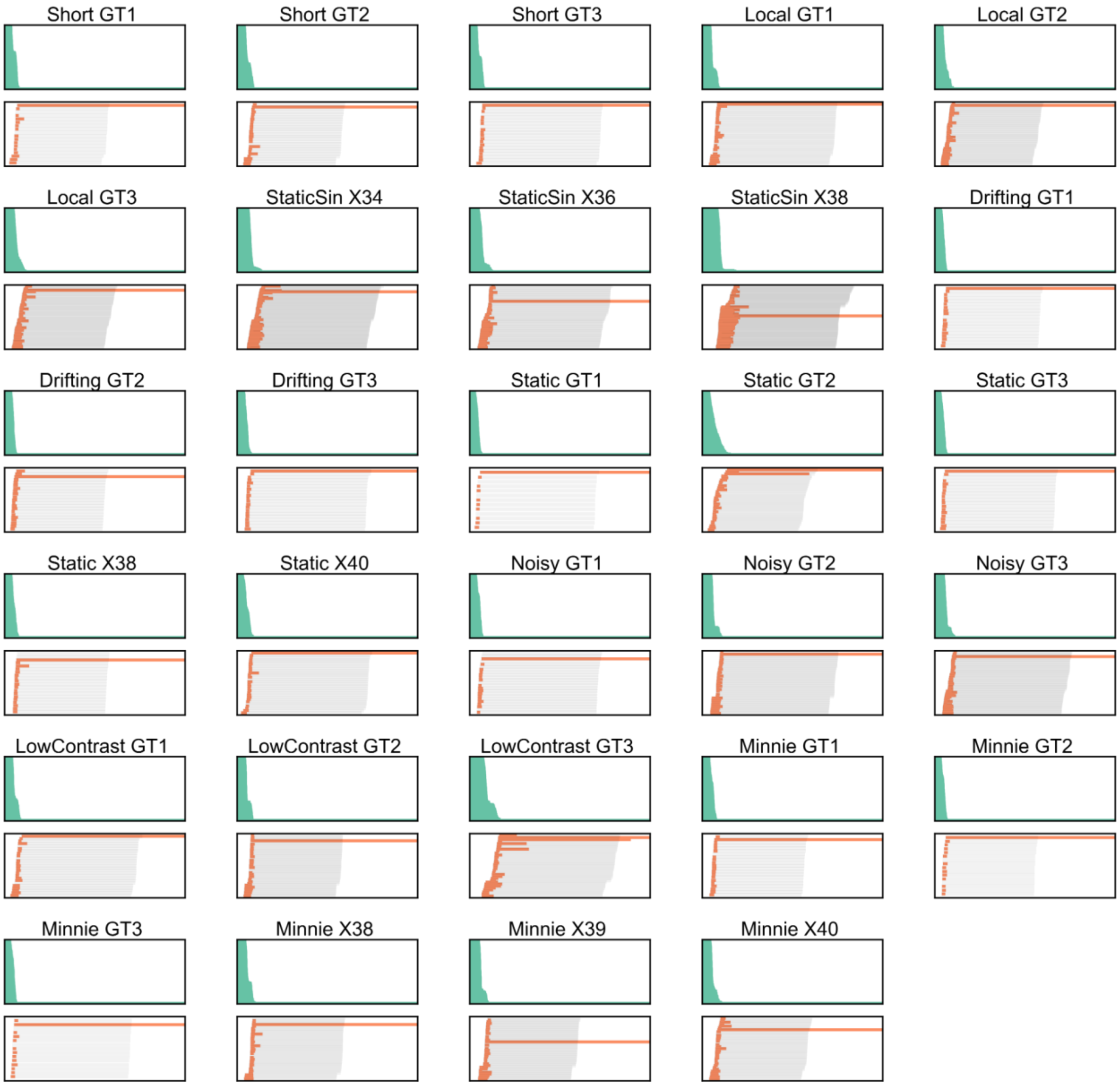

Figure 6: **All the barcodes computed with the geodesic metric when all cells are included as shown in Figure 2.**
The titles denote the stimulus type and the name of the mouse. The gray shading corresponds to the estimated persistence threshold at which a feature can be determined as significant.
